# A brief report on juvenile amyotrophic lateral sclerosis cases in the United States National ALS Registry: 2010–2018

**DOI:** 10.1080/21678421.2023.2264922

**Published:** 2024-01-23

**Authors:** JAIME RAYMOND, JASMINE BERRY, EDWARD J. KASARSKIS, THEODORE LARSON, D. KEVIN HORTON, PAUL MEHTA

**Affiliations:** 1 Agency for Toxic Substances and Disease Registry/Centers for Disease Control and Prevention, Atlanta, GA, USA; 2 Department of Neurology, University of Kentucky College of Medicine, Lexington, KY, USA

**Keywords:** amyotrophic lateral sclerosis, ALS, juvenile ALS, motor neuron disease

## Abstract

Juvenile ALS (jALS) is a rare form of ALS, defined as symptom onset before age 25. This report describes the demographic characteristics of confirmed and likely jALS cases in a large cohort of ALS patients ascertained in the National ALS Registry (Registry) from 2010 to 2018. Patients in the Registry must be at least 18 years of age. Of the 44 identified patients, 37.8% were diagnosed at age 24, were more likely to be nonwhite (54.5%), male (79.5%), and live in the Midwest or Northeast regions (54.5%) of the US. Some 68.9% of the jALS cases were received from federal administrative databases, and 16% came from the web portal only. Demographic characteristics for jALS cases in the Registry differed from previous publications examining ALS cases for all adults. More research is needed to better understand risk factors contributing to jALS, which could lead to earlier diagnosis and therapeutic interventions.

## Introduction

Amyotrophic lateral sclerosis (ALS) is a progressive and fatal neuromuscular disease with the majority of patients dying within 2–5 years of receiving a diagnosis (1). Familial ALS, a heredity form of the disease, accounts for 5–10% of cases, whereas the remaining sporadic cases have no clearly defined etiology (1). Previous reports show most ALS cases in the United State (U.S.) are White males with an onset between 60 and 69 years of age (2). A small percentage of ALS cases present with symptoms before age 25 and are classified as juvenile ALS (jALS) cases (3). This report describes the demographic characteristics of confirmed and likely jALS cases ascertained in the National ALS Registry between October 19, 2010 – December 31, 2018.

## Methods

In 2008, the U.S. Congress passed the ALS Registry Act, which authorized the creation and maintenance of the National ALS Registry (Registry) by the Centers for Disease Control and Prevention’s (CDC) Agency for Toxic Substances and Disease Registry (ATSDR). Data collection began in 2010 (4). The Registry’s goals and methods have been described in detail in previous publications (5). ALS patients in the U.S. are identified from large national administrative databases by using an updated algorithm with elements such as the ICD code for ALS, frequency of visits to a neurologist, and prescription drug use, which categorizes individuals in the Registry as “confirmed ALS,” “likely ALS,” “undetermined ALS,” and “not ALS” (6). Only confirmed and likely ALS are considered cases and entered into the Registry. ALS cases are also identified via a secure web portal that enables person with ALS to enroll directly into the Registry. Cases from both sources are then merged and deduplicated. While jALS is defined as onset before 25 years (7), the Registry does not have access to symptom onset for patients, this analysis will define jALS as a confirmed or likely ALS before age 25. Currently, the Registry does not collect data from patients under the age of 18 due to CDC Institutional Review Board policies. We tabulated Registry data between 2010 and 2018 based on patient factors: sex, race, age, and census region.

## Results

Forty-four cases were identified in the National ALS Registry from October 19, 2010 to December 31, 2018 as confirmed or likely ALS before age 25. Of those, 17 patients (38.6%) were diagnosed at age 24 (Table 1). The northeast census region had the highest percent of jALS patients in the Registry at 29.5%. jALS patients were more likely to be nonwhite (54.5%) and males had more than 3 times as many cases compared to females (79.5% vs 20.5%) (Table 1). The crude and age adjusted prevalence rates remained steady from 2015 to 2018 with a slight increase in the age-adjusted prevalence from 0.21 to 0.24 per 100,000 persons during the interval (Table 2).

**Figure 1.**
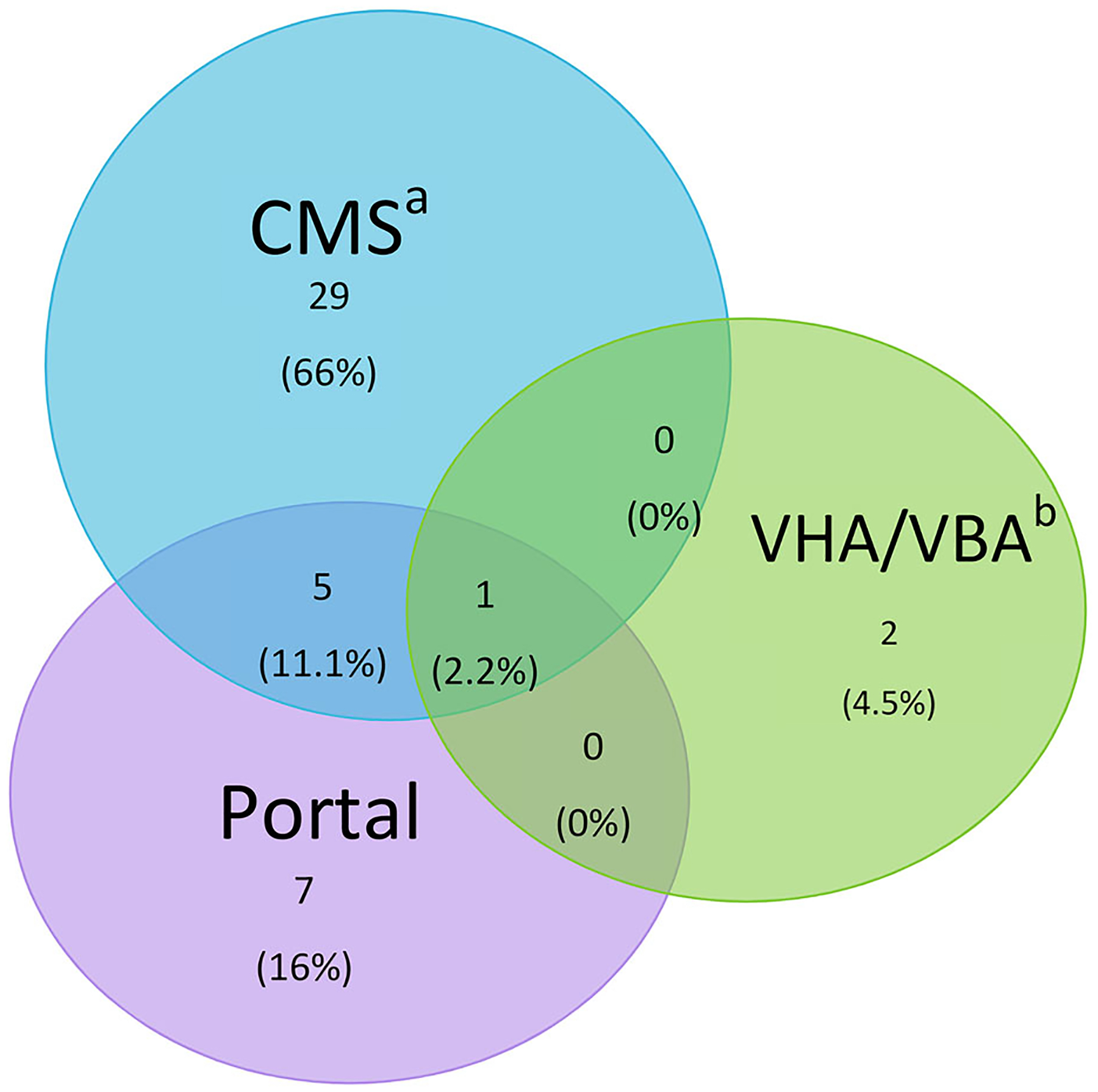
Prevalent jALS case ascertainment overlap in the National ALS Registry, 2011–2018. a – Center for Medicaid/Medicare Services b – Veterans Heath Administration/Veterans Benefits Administration

To determine the data sources of the jALS cases, we collected the source for patients entering the Registry as a confirmed or likely case (Figure 1). For confirmed and likely cases in the Registry, 69% came from (Centers for Medicare & Medicaid Services) CMS followed by 25% from the web portal. Some 12% of patients entered the Registry in both the web portal and CMS. Six percent came from Veterans Health Administration/Veterans Benefits Administration (VHA/VBA).

## Discussion

The 44 cases identified by the Registry represents a small fraction of the sporadic cases which is expected. The Registry is missing cases especially from younger ALS cases (2,8). These cases however have a racial and ethnic pattern that was not expected and is inverse to previous demographics identified by the Registry as a whole (i.e., non-Hispanic and White) (2,5). Additional research is needed to understand this phenomenon.

Another interesting finding was 3 cases were found from VHA/VBA. This represents patients who previously served in the military and were diagnosed with jALS during their service or shortly thereafter. jALS is a rare subset of ALS and is most likely associated with gene mutations such as fused in sarcoma (FUS) and ALS2 (9), FUS being more common (10). Additional research is needed to determine likely causes of juvenile ALS cases in the United States. If the Registry could add patients less than 18 years of age, we could continue to study this ALS population.

## Limitations

The following are limitations of our analyses. First, we do not include cases under the age of 18 and this may represent a nominal undercount. Second, there is no genetic testing available for these cases from the National ALS Biorepository, since these cases did not elect to participate. Next, we did not have clinical characteristics on these patients to further investigate the juvenile cases. Last, our small size of 44 jALS cases makes it difficult to make definitive conclusions. These factors contribute to our limited knowledge of juvenile ALS.

## Figures and Tables

**Table 1.** Demographics of jALS patients (*N* = 44) based on year the patient became a confirmed or likely case, National ALS Registry, 2010–2018.

	Overall N=44 %)
**Age (years)** ^**a**^	
< 24	27 (61.4)
24	17 (38.6)
**Race/Ethnicity**	
Non-Hispanic White	20 (45.5)
Other Race/Ethnicities	24 (54.5)
**Sex**	
Male	35 (79.5)
Female	9 (20.5)
**Census Region**	
Midwest	11 (25.0)
Northeast	13 (29.5)
South	7 (15.9)
West	7 (15.9)
Unknown	6 (13.6)

**Table 2.** Age-adjusted rates of combined likely and confirmed cases and estimated prevalence for jALS patients in the National ALS Registry, 2015–2018.

Year	Cases	Population	Crude Rate*	Age-Adjusted Rate*
2015	34	30,796,315	0.18	0.21
2016	35	30,250,180	0.19	0.22
2017	34	29,947,400	0.19	0.21
2018	38	30,705,359	0.21	0.24

* per 100,000.
